# NextGEM: Next-Generation Integrated Sensing and Analytical System for Monitoring and Assessing Radiofrequency Electromagnetic Field Exposure and Health

**DOI:** 10.3390/ijerph20126085

**Published:** 2023-06-08

**Authors:** Nikolaos Petroulakis, Mats-Olof Mattsson, Panos Chatziadam, Myrtill Simko, Andreas Gavrielides, Andrianos M. Yiorkas, Olga Zeni, Maria Rosaria Scarfi, Eduardo Soudah, Ruben Otin, Fulvio Schettino, Marco Donald Migliore, Andreas Miaoudakis, George Spanoudakis, John Bolte, Erdal Korkmaz, Vasileios Theodorou, Eleni Zarogianni, Susanna Lagorio, Mauro Biffoni, Andrea Schiavoni, Mauro Renato Boldi, Yuri Feldman, Igal Bilik, Anna Laromaine, Martí Gich, Marco Spirito, Maryse Ledent, Seppe Segers, Francisco Vargas, Loek Colussi, Mathieu Pruppers, Dan Baaken, Anna Bogdanova

**Affiliations:** 1 Institute of Computer Science, Foundation for Research and Technology-Hellas (FORTH-ICS), 70013 Heraklion, Greece; 2 SciProof International AB, 83158 Ostersund, Sweden; 3 eBOS Technologies Limited, Nicosia 2322, Cyprus; 4 Institute for Electromagnetic Sensing of the Environment, Consiglio Nazionale delle Ricerche (CNR-IREA), 80124 Napoli, Italy; 5 International Centre for Numerical Methods in Engineering (CIMNE), 08034 Barcelona, Spain; 6 Department of Electrical and Computer Science Engineering, University of Cassino and Southern Lazio, 03043 Cassino, Italy; 7 Sphynx Analytics Limited, Nicosia 2012, Cyprus; 8 Research Group Smart Sensor Systems, The Hague University of Applied Sciences, 2628 AL Delft, The Netherlands; 9 Centre for Sustainability, Environment and Health, National Institute for Public Health and the Environment (RIVM), 3720 BA Bilthoven, The Netherlands; 10 Intracom Telecom, 19002 Peania, Greece; 11 Italian National Institute of Health, 00161 Rome, Italy; 12 Telecom Italia Spa, 20123 Milan, Italy; 13 Department of Applied Physics, The Hebrew University of Jerusalem, Jerusalem 91904, Israel; 14 Department of Electrical and Computer Engineering, Ben Gurion University of the Negev, Beer Sheva 8410501, Israel; 15 Institut de Ciència de Materials de Barcelona, Consejo Superior de Investigaciones Científicas (ICMAB-CSIC), 08193 Barcelona, Spain; 16 Department of Microelectronics, Delft University of Technology, 2628 CN Delft, The Netherlands; 17 Sciensano, 1050 Elsene, Belgium; 18 Ministry of Health, 28014 Madrid, Spain; 19 Dutch Authority for Digital Infrastructure, 9700 AL Groningen, The Netherlands; 20 Institute of Medical Biostatistics, Epidemiology and Informatics, University Medical Center of the Johannes Gutenberg-University Mainz, 55131 Mainz, Germany; 21 Institute of Veterinary Physiology, University of Zurich, 8006 Zurich, Switzerland

**Keywords:** radio frequency (RF), electromagnetic field (EMF), communication engineering and systems telecommunications, biological effects, occupational health, public and environmental health

## Abstract

The evolution of emerging technologies that use Radio Frequency Electromagnetic Field (RF-EMF) has increased the interest of the scientific community and society regarding the possible adverse effects on human health and the environment. This article provides NextGEM’s vision to assure safety for EU citizens when employing existing and future EMF-based telecommunication technologies. This is accomplished by generating relevant knowledge that ascertains appropriate prevention and control/actuation actions regarding RF-EMF exposure in residential, public, and occupational settings. Fulfilling this vision, NextGEM commits to the need for a healthy living and working environment under safe RF-EMF exposure conditions that can be trusted by people and be in line with the regulations and laws developed by public authorities. NextGEM provides a framework for generating health-relevant scientific knowledge and data on new scenarios of exposure to RF-EMF in multiple frequency bands and developing and validating tools for evidence-based risk assessment. Finally, NextGEM’s Innovation and Knowledge Hub (NIKH) will offer a standardized way for European regulatory authorities and the scientific community to store and assess project outcomes and provide access to findable, accessible, interoperable, and reusable (FAIR) data.

## 1. Introduction

While emerging technologies that use radiofrequency electromagnetic fields (RF-EMF, 100 kHz–300 GHz), particularly in telecommunications, are vital for modern life, there is an increasing consideration of the possible adverse effects on human health and the environment, which may be potentially exacerbated by aggregation of different types of RF-EMF signals. Some concerned citizen groups perceive fifth-generation telecommunication systems (5G; 5G New Radio; 5G NR) as a more significant threat to public health than previous-generation systems. The exposure guidelines and standards issued by the International Commission for Non-Ionizing Radiation Protection (ICNIRP) and the International Committee on Electromagnetic Safety of the Institution of Electrical and Electronic Engineers (ICES-IEEE) are set to prevent the occurrence of such adverse effects. These guidelines are based on comprehensive reviews of the relevant scientific literature, providing exposure reference levels and basic restrictions for workers and the general population; the latter includes an additional safety factor to account for vulnerable groups. Regarding occupational exposures, the European Union (EU) follows the ICNIRP guidelines [1] (Directive 2013/35/EU [2]), which are in force in all Member States. As for exposure to the general public, the EU published a recommendation (1999/519/EC [3]) for exposures to EMF (0 Hz to 300 GHz), with limits derived from the ICNIRP 1998 guidelines [1]. However, due to the non-binding nature of the recommendation and the different types of EU legislation [4], the related policies vary across European countries [5]. In addition, the exposure limits established by the ICNIRP are based on a thermal threshold. However, there is a debate about the adequacy of these limits and the need to include non-thermal effects for which the results are currently inconclusive. The limits are based on solid and conclusive evidence, while non-thermal effects are not conclusive and not widely accepted by the scientific community.

Considering all the above, the adoption of new telecommunication technologies such as 5G requires extensive investigations regarding the potential causal effects between RF-EMF and health to address the following needs:

- Updated appraisal of the scientific evidence regarding possible links between RF-EMF and health effects for (i) the general public, (ii) workers, and (iii) vulnerable groups to aid public authorities in implementing evidence-based policies, risk assessment, and communication.
- Quality criteria, standards, and methodologies (i) to assess RF-EMF exposure and health effects, and (ii) for biological investigations fulfilling the 3R (Replace, Reduce, and Refine) standards.
- Understanding interaction mechanisms between RF-EMF and biological systems, including combined exposures with other agents and multiple signals.
- Adequate scientific communication to improve awareness among authorities, employers, and citizens, counteracting RF-EMF misinformation.

**Figure 1:**
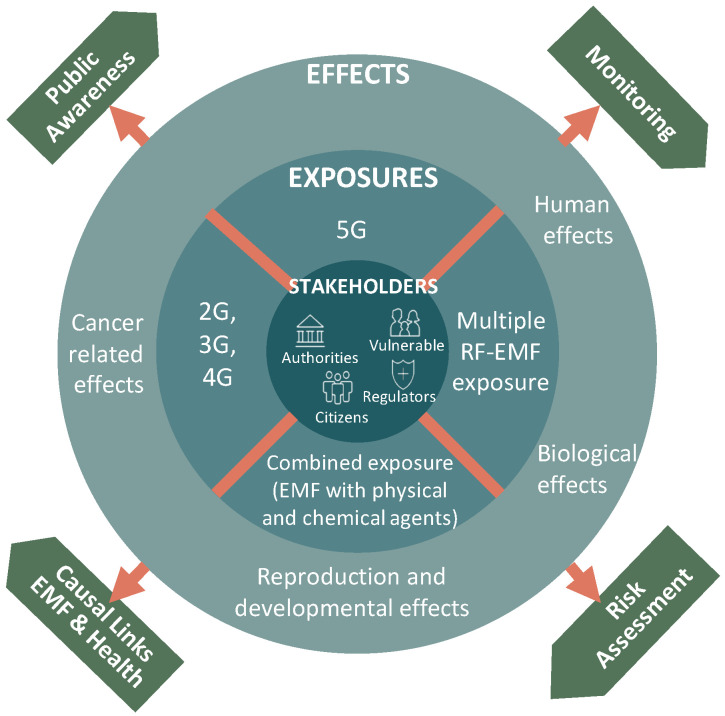
NextGEM approach.

The NextGEM project will provide a framework (see Figure 1 for an overview of the NextGEM approach) for generating health-relevant scientific knowledge and data on new scenarios of exposure to RF-EMF in multiple frequency bands (“NextGEM: Next-Generation Integrated Sensing and Analytical System for Monitoring and Assessing Radiofrequency Electromagnetic Field Exposure and Health” is a 4-year research and innovation project (1 July 2022–30 June 2026 funded under Horizon Europe GA 101057527, www.nextgem.eu (accessed on 1 March 2023)). A variety of experimental studies will be carried out at different levels of non-thermal exposures to assess the occurrence of potential effects of RF-EMF alone or in combination with other agents during short- and long-term exposures. Based on the literature reviews, including papers published by other consortia and the work carried out in the project, an exploratory risk quantification, in the case of causal effects of RF-EMF alone or in co-exposure, will be performed. NextGEM will develop and validate tools for evidence-based risk assessment. NextGEM will also implement the Innovation and Knowledge Hub (NIKH) for RF-EMF and Health, offering a standardized way for European regulatory authorities and the scientific community to store and assess project outcomes and provide access to FAIR data. All the experimental results from NextGEM will be available in the knowledge base of NIKH platform that will be integrated in the NextGEM website along with scientific results (public deliverables, publications, guidelines, policy recommendations) and other project results such as brochures, flyers, video clips etc. The final goal is to promote a healthy living and working environment, especially for vulnerable groups, under safe RF-EMF exposure conditions, trusted by people, and in line with the regulations and laws issued by public authorities.

The vision of the NextGEM project to assure safety for EU citizens when employing existing and future EMF-based telecommunication technologies is presented in this article and organized as follows: Section 2 focuses on the related activities and NextGEM’s advancement of existing knowledge. Next, Section 3 presents NextGEM’s methodology under specific directions. Section 4 presents the identified case studies to evaluate the investigated research and developed directions. Finally, Section 5 concludes this article.

## 2. Related Activities

NextGEM performs several activities, including measurements and modeling of 5G exposure patterns (i.e., FR1: frequency band 1 (below 7.125 GHz); FR2: frequency band 2 (above 24.250 GHz)) with the respective RF-EMF modeling tools. In addition, the physics of the processes initiated by exposure will be studied and experimental in vitro and in vivo and human studies will be carried out. Thus, NextGEM evaluates possible causal risks by conducting umbrella reviews of published systematic reviews and meta-analyses and performing a health risk assessment. The ambition of NextGEM with regard to other related works is presented in the next subsections.

### 2.1. 5G Exposure Patterns

5G New Radio (NR) differs from earlier generations of wireless technologies (e.g., frame duration, bandwidth, and power allocation) in its use of massive multiple input multiple output (Ma-MIMO) beam-forming and in the use of millimeter waves (i.e., FR2) to support multiple usage scenarios [6,7]. The current deterministic approaches in exposure distribution for passive antennas used in earlier generations of mobile communication are different from the actual exposure to Ma-MIMO 5G antennas. Ma-MIMO antenna technology has introduced a degree of freedom in the management of exposure distribution. Based on that, the possibility to shape and direct emissions only in the direction where service is required allows optimization of the coverage and power usage with a consequent reduction of total exposure. Under these conditions, the exposure is present and distributed per the requested services. This makes power distribution estimation more challenging, necessitating creative developments of statistical features suitable for power distribution estimates in time and space. NextGEM accomplishes necessary and much sought-after improvements in field-level assessment in real scenarios by developing a maximum power extrapolation technique and a suitable statistical model in both single user (SU) MIMO and multiple user (MU) MIMO cases.

### 2.2. RF-EMF Modelling Tools

RF-EMF modelling tools are able to compute the fields generated by radiating sources; however, they may exhibit limitations in modeling capabilities, availability, and computational performance. For instance, finite-difference time-domain (FDTD) tools require box-like discretization of the geometry, which can induce modeling errors on generally curved geometries. There are also techniques that allow the management of surfaces not aligned to the grid. Considering the changing exposure landscape, it is urgent to develop a framework of open-source tools to fill the gaps not covered by the commercial software by adding new multi-physics models (high-performance computing (HPC)-platform-compatible) to improve accuracy and detail.

NextGEM will develop next-generation, open-source computational tools for RF-EMF environmental levels and body-absorbed energy measurements. New numerical algorithms will be tested and validated to improve numerical efficiency and accuracy. New interfaces (multiple wave planes with human-body geometry and a full-wave EM solver) and capabilities (coupling EM with other physical models) will be developed, further advancing RF-EMF modeling in complex environments. These new algorithms will be implemented in ERMES [8]. ERMES is an open-source finite element code in the frequency domain that implements in C++ a simplified version of the weighted regularized Maxwell equation method. This finite element formulation produces well-conditioned matrices which can be solved efficiently with low-memory-consuming iterative methods. Furthermore, thanks to the null kernel of its differential operator, it can operate distinctly in quasi-static and high-frequency regimens.

### 2.3. Physics of the Processes Initiated by Exposure of Living Cells to RF-EMF

Dielectric dispersion is a dissipative (i.e., thermal) phenomenon. However, the non-thermal interaction of RF-EMF with biological systems may be based on dielectric dispersion as the permittivity decreases with increasing frequency [9]. For RF-EMF non-thermal effects of mobile communication carrier frequencies, the targets may include fast mobility of amino acids, lipids, and nucleotides, as well as proton hopping (3G/4G operation frequencies) or water (5G) [10]. As 3G–5G RF-EMFs have a minor effect on the dipole or charged groups’ mobility, it is possible that the envelope of the modulated carrier frequencies affects the cells. Specifically, in this case, the action of the signal is expected on the organelles, membranes, and nuclei due to the slow migration of ions or deformation of ion clouds at the membrane surfaces, or polarization of cellular membranes [11]. A progressive increase in carrier frequencies used for telecommunication results in the corresponding reduction of the penetration depth through the human body. Although human skin penetration depth at 3G–5G operation frequencies (0.5–60 GHz) affects only the skin dermis layer [12], blood may respond to the RF-EMF when passing through the dermal microvasculature and thus transducing the signal to the peripheral tissues [13]. Indeed, previous studies indicate non-thermal effects of acute exposure to RF-EMF on erythroid precursors and red blood cells (RBCs) [14]. Significant knowledge advancement regarding the possible impact of RF-EMF on (i) hemoglobin function, (ii) the state of hydration shells and free water, and (iii) membrane properties will be accomplished in NextGEM. Frequency dependencies, response kinetics, and duration, as well as tissue-specificity, will be studied. Such a mechanistic understanding is crucial to understanding if non-thermal RF-EMF effects are possible and, if so, under which circumstances.

### 2.4. In Vitro and In Vivo Investigations

A large amount of in vitro and in vivo studies are available on the effects of 2G–4G RF-EMF [15,16], while 5G has not yet been investigated. In the current published literature, in most cases, no effect was detected, and when present, it was due to a temperature increase. However, some studies suggested that non-thermal exposure can affect a variety of biological functions, including the expression of several genes [15], although these findings have not been confirmed in other independent investigations [17]. Overall, results are often contradictory, and well-performed mechanistic studies, using an intervention in biological processes investigating specific biochemical and molecular events and molecules, are needed [18]. The majority of the studies were performed using one single frequency and not considering co-exposures using different RF-EMFs or other agents, which could reveal possible synergistic, additive or protective effects of different factors. Furthermore, a substantial part of the studies suffers from severe quality deficiencies [16]; therefore, these studies have limited use for risk assessment. For a better understanding of the biological effects and molecular mechanisms of 3G, 4G, and 5G RF-EMF exposure in realistic scenarios, including combined exposure (with other agents) and multiple exposure (with different RF-EMF frequencies and signals), in vitro (cell lines and primary human cells), in vivo (*C. elegans*), and ex vivo (human RBC and lymphocytes) studies will be conducted based on harmonized exposure and experimental protocols between the different groups. Such studies focusing on complex exposure conditions have not been previously performed and go significantly beyond the state of the art (SoTA). The focus is on effects related to carcinogenesis and reproductive toxicity and Hb conformation in human RBC. Different -omics approaches will also be used to identify genes and proteins as potential exposure marker candidates. Candidate genes identified will be further investigated in an ex vivo exposure study on lymphocytes and in an exploratory human study. The obtained results will be the foundation for hazard and risk assessments within the NextGEM project, allowing these activities to be performed more accurately.

### 2.5. Human Studies

The micronucleus (MN) test can investigate human genetic damages on, e.g., exfoliated buccal cells. Several studies applied this technique to explore the potential adverse effects of mobile phone RF-EMF, but the results were contradictory [19,20]. Most of the studies have several shortcomings that preclude firm conclusions [21]. Furthermore, the lack of knowledge about the characteristics of MN and other nuclear abnormalities in oral cells also limits the interpretation of the results. Results bringing the knowledge significantly beyond SoTA regarding genetic damage due to RF-EMF exposure in humans will be obtained from studies on MN formation and gene expression in exfoliated buccal cells from heavy and light mobile phone users. Different techniques will be applied for the definition of both groups to prevent the limitations identified in previous studies as much as possible. A pilot study on healthy volunteers will also be conducted (double-blind) after exposure to 5G carrier frequencies and modulation envelopes to detect potential acute effects on physiological cell parameters in human blood cells (RBC, lymphocytes). In this pilot study, a container set up to reduce the levels of environmental electromagnetic fields (from the building and outside) located in ISSeP-Liège (Belgium) will be used where on the right side of the container is the test room for the volunteers and on the left side is the technical equipment, including the test instruments and the computer that manages the exposures.

### 2.6. Umbrella Review of Neoplasia Risks from RF-EMF Exposure in Humans

The methodology of systematic reviews (SR) of environmental health topics, including the harmonization of the currently disparate approaches for assessing the quality and weight of evidence, is undergoing major improvements. Consensus-building efforts have recently led to the publication of a set of “Recommendations for the conduct of systematic reviews in toxicology and environmental health research (COSTER)” [22]. Several SRs of cancer hazards from exposure to RF-EMF are in progress, promoted by the WHO [23] and other entities [24], including a planned re-evaluation of the carcinogenicity of RF-EMF by the IARC [25]. However, no epidemiological study has investigated cancer risks and how they relate to individual integrated exposure from multiple RF-EMF sources.

To provide the integrated exposure and carcinogenesis data for evidence-based risk assessment, NextGEM will perform umbrella reviews of SRs and meta-analyses of human observational studies of neoplasm risks from exposure to RF-EMF. Umbrella reviews can provide the highest quality of evidence if performed and interpreted correctly. This novel approach to RF-EMF health effects studies goes significantly beyond the state of the art. The available SRs will be compared in terms of transparency, fidelity to predefined protocols, utility in answering the scientific question(s) of interest, and credibility of their findings, using reference standards and expert judgment. The utility and credibility of an SR depends on the adequacy of the tools used to assess the most likely sources of bias in a given body of evidence and on the soundness of the conceptual framework used to appraise and integrate the relevant evidence streams. NextGEM possesses the methodological and subject-matter expertise required for a critical summary of findings from SRs of epidemiological and experimental studies of the effect of RF-EMF exposure on cancer and reproductive outcomes and will focus on these hazards [26,27].

### 2.7. Health Risk Assessment

A risk assessment needs to include exposure assessment, hazard identification, and characterization, including knowledge about the mode of action that underpins the risk assessment process. Although many institutions perform risk assessment [28], there is no standard procedure for evidence-based risk assessment. The European Commission suggests a “Weight of evidence” (WoE) approach [29] where evidence from epidemiological and experimental human in vivo, in vitro, and in silico studies are integrated for an overall risk assessment. Challenges for RF-EMF risk assessment include that risk assessment generally takes exposure guidelines into account, where primarily acute effects are the only ones considered. Risk assessment for exposure situations characterized by long-term and combined exposures and/or co-exposures with other agents do not exist.

For the first time, integrated risk assessment protocols will be provided, including exposure situations characterized by long-term exposures, combined exposures to different RF-EMFs, and co-exposures with other agents. NextGEM will ensure this by pronounced stakeholder involvement, integrating novel exposure and dosimetry methodologies for RF-EMF exposure assessment, and identifying health-relevant markers in high-quality experimental bio-effects studies. A unique risk assessment tool will be developed in collaboration with and for use by different stakeholders and validated in case studies.

## 3. Methodology

### 3.1. The NextGEM Project Methodological Approach

The NextGEM framework can efficiently integrate all technical enablers of the project and encompass real-time monitoring and historical and experimental data.

- **Sensing and data source:** Data for RF-EMF exposure assessment will be collected from the literature and/or experimentally measured in different real-life scenarios through the case studies conceived and developed during the project. As regards the new generation communication networks, novel measurement paradigms aim to be developed together with wearable sensing devices suitable for the 5G mm wave band.
- **Analytics and experimentation:** The combination of multiple approaches from real-life case scenarios, exposure assessment, and umbrella reviews of epidemiological data in combination with in vitro, ex vivo, and in vivo experiments elucidates possible biological and health effects of RF-EMF interactions and their underlying mechanisms.
- **Applications, tools, and services:** NextGEM will provide novel exposure assessment devices and protocols as well as risk assessment tools, integrated into the *NIKH*, representing the main key exploitable outcomes.

### 3.2. RF-EMF Exposure Assessment Modeling and Measurements

**Figure 2:**
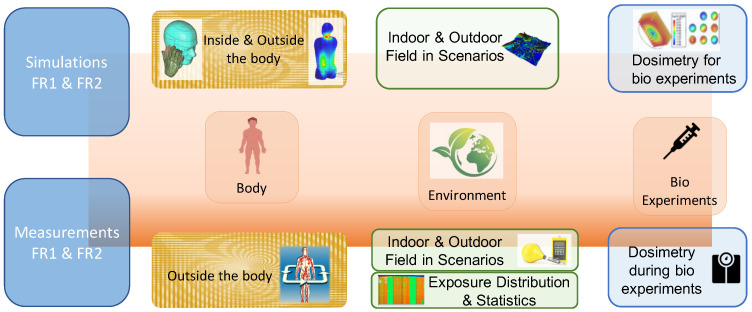
Multi-scale approach to RF-EMF exposure assessment.

The RF-EMF exposure assessment includes both modeling and measurements of the experimental setup (dosimetry), RF-EMF levels inside and outside the human body, and environmental RF-EMF levels in indoor and outdoor scenarios (Figure 2).

**Multi-Scale Model-Based Assessment of RF-EMF Exposure in the Human Body:** The scope of this activity is a multiscale approach to RF-EMF exposure assessment by simulation and/or measurements from the small (cells and small organisms) to medium (human body) and large scale (cities, buildings). Initially, the unperturbed RF-EMF distribution generated by the radiating source will be calculated (or measured) at every point of interest in the indoor/outdoor environment (city, workplace, school, home). This information will be used as input to the RF-EMF software tool for the exposure conditions in the experiments to calculate the fields outside/inside a human body. The various simulations and experiments will take place in parallel and will be used bidirectionally to improve computational models and experimental conclusions to compare the results. The RF-EMF software will be used with exposure source properties, the radiated region’s CAD model, the human body’s electromagnetic properties, and the human body’s CAD model.

**Exposure Compliance Assessment and Environmental Protection:** RF-EMF exposure assessment is particularly challenging in 5G due to its high flexibility with respect to the antenna technologies employed, such as beamforming capabilities and/or different MIMO implementations, as well as its sophisticated energy-efficient signaling structure. NextGEM will develop novel measurement techniques for different antenna technologies (such as continuous beamforming, a grid of beams, SU-MIMO, MU-MIMO, and Ma-MIMO, based on maximum power extrapolation (MPE)). MPE techniques have been proposed and successfully applied in past-generation cellular systems but require a generalization from a “passive” determination of what is on air in a certain moment to an “active” measurement forcing the system under test to assume the most suitable configuration. In NextGEM, novel statistical models will be developed for realistic exposure assessment in both indoor and outdoor scenarios measurements. A possible approach [30] relies on the normalized average power pattern (NAPP), which seems particularly suitable to compare different antenna technologies and scenarios, and is easy to integrate with stochastic models of user distributions, as well as with ray-tracing algorithms. Channel power measurements will be performed with a spectrum analyzer, whereas measurements in the code domain will be performed with a signal analyzer with 5G NR decoding capabilities. Both directive and omnidirectional antennas will be used. Both line of sight (LOS) and non-LOS will be tested with one or more user devices.

**RF-EMF exposure paradigms and self-monitoring:** 5G NR technology makes intensive use of MIMO technologies, and the user’s exposure is related to the EM coupling in the body. For these reasons, a body-worn portable exposure meter can mimic real scenarios obtaining reliable data to monitor RF-EMF exposure. The NextGEM project will develop multiple body-wearable portable exposure meters to be employed in real-life use-case scenarios. To achieve this novel wearable sensing device, the current sensing node, operating at 27.5 GHz, will be redesigned using planar antennas with plastic lenses (easier to integrate into a wearable device), providing higher spatial selectivity. The newly developed sensor will be tested in the ADome TU Delft characterization facility, allowing the identification of the critical electrical specification of the sensor and adequate design of the sensor-carrying vest with full spatial coverage. The single sensors and the vest’s first prototypes will be tested in the TU Delft Green Village. In this campus location, 5G FR2 bands are licensed to the university for real use-case testing scenarios. The devices and the vest will then be fully characterized inside this test location with known excitation signals for being employed in the measurement campaign.

### 3.3. Experimental Studies

**Figure 3:**
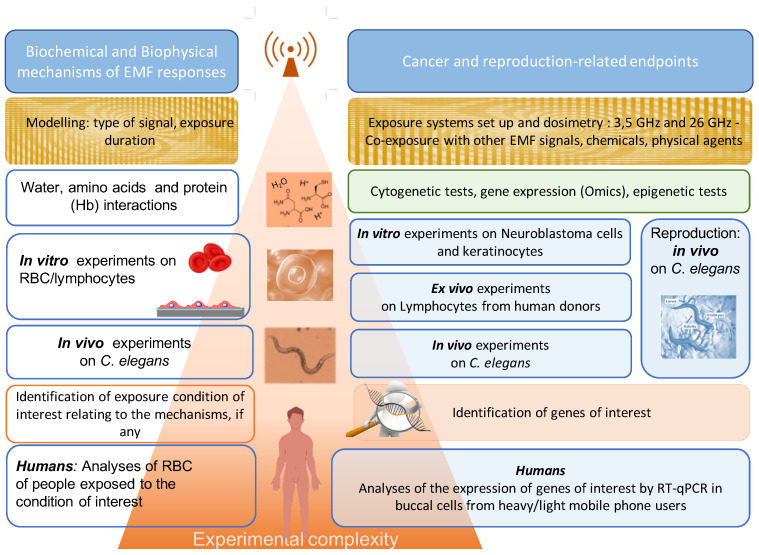
Overall strategy for biological investigations.

NextGEM aims to collect robust and reliable data by combining different experimental approaches: in vitro studies on human cell lines, in vivo studies on the model organism Caenorhabditis elegans (*C. elegans*), and ex vivo exposure studies on human cells (lymphocytes). Certain in vitro studies will be carried out at more than one lab for replication purposes. Cell models used for experimental studies have been chosen because of the existence of standardized and widely recognized assays (buccal epithelium cells, lymphocytes, neuroblastoma cells), appropriateness for studies of the poorly penetrating 5G signals (keratinocytes), and relative simplicity in the organization and lack of morphological variation (RBC), as can be seen in Figure 3. The experimental studies in NextGEM focus on cancer-related endpoints, reproduction and development, and finally on different biophysical and biochemical mechanisms, as will be described below. All experiments are going to be performed taking into consideration environmental factors such as noise, vibrations, and background magnetic fields and will eliminate these factors.

**In vitro studies on mammalian cell lines:** Human neuroblastoma and keratinocyte cell models will be used to investigate the effect of exposure to 4G and 5G signals, given alone, as multiple frequencies, and combined with other agents, to evaluate cancer-related endpoints. Numerical and experimental dosimetry techniques will be employed to design and characterize new exposure systems and/or optimize existing ones and to calculate the dosimetry parameters (specific absorption rate (SAR), electric field, power density) in the sample. The design of the applicator device will be driven by efficiency and SAR uniformity criteria, ensuring the control of environmental (temperature, humidity, CO${}_{2}$) parameters according to the recommendations for good laboratory practice (GLP). For dose homogeneity (numerically evaluated through the variation coefficient, i.e., the ratio between standard deviation and average SAR), different solutions have to be adopted in the sub-mmWave band [31] and in the lower frequency band [32]. Several solutions (waveguides, transverse EM cell, radial transmission line, wire patch cell, reverberating chambers, etc.) will be considered. Short (1 to 24 h) and long (3 weeks) exposure durations will be applied, according to the biological systems used and the endpoints measured, at different SAR values, to investigate the effect of 4G (1950 MHz) and 5G (3.5–26.5 GHz) signals. Oxidative stress, apoptosis, and cell cycle progression will be analyzed (flow cytometry, molecular biology techniques). Genomic instability will be evaluated by micronucleus (MN) and Comet assays. Transcriptomics and epigenetic tests will be performed on selected conditions based on the outcomes of the above-mentioned tests. Two conditions will be investigated on human neuroblastoma cells (apoptosis, cell cycle progression) to test multiple RF-EMF signals: (a) 1800 MHz GSM, 1950 MHz UMTS, and 2.4 GHz WiFi signals and (b) 3.5 GHz and 2.4 GHz WiFi. To address the effect of combined exposures, 4G exposure will be performed in combination with menadione (human neuroblastoma cells) and 5G exposure in combination with UV-B light (human keratinocytes).

**In vivo studies on** ***C. elegans*****:** *C. elegans* will be employed as an in vivo simple model to evaluate cancer-related endpoints and perform genetic screening. Furthermore, reproduction in the parental and second generation will be also assayed. The exposure system is similar to the one adopted for in vitro studies. Oxidative stress and genetic screening will be evaluated on wild-type *C. elegans* (N2) in vitro with fluorescent markers. For reproduction assays, synchronized nematodes will be exposed at different developmental stages—eggs and L1 to L4 larval stages. Growth, egg and organism development, and motility parameters will be assessed by imaging processing. Gene expression will be assayed as in in vitro and ex vivo experiments.

**Ex vivo studies on** ***lymphocytes***** from human donors:** *Lymphocytes* from human donors will be exposed to 5G signals (3.5 GHz), alone and in combination with other agents, to analyze the expression of a selection of cancer-related genes. The exposure system and dosimetry is similar to the one adopted for in vitro studies on keratinocytes. Ex vivo exposure will be performed on *lymphocytes* collected from healthy human donors, with the same exposure conditions adopted for the short-term in vitro studies. Combined exposures will be carried out on lymphocytes with ethyl methanesulfonate (EMS)/menadione genotoxicants. The comet assay will be applied to evaluate the induction of DNA damage. Gene expression and epigenetic tests will be applied to selected conditions based on the Comet assay results.

**Biological effects of RF-EMF exposure in humans:** Buccal cells from self-reported heavy and light mobile phone users will be used to investigate the expression of genes identified in in vitro and ex vivo studies. Self-reported mobile phone users will be selected based on the amount of time calling with the mobile phone at the ear level. Information will be obtained related, e.g., to the type of phone used in the last 10 years and the behavior when calling, to estimate SAR values at the inner cheek level (dosimetry modeling). Moreover, actual measurements are envisaged to confirm the classification in both groups. Buccal cells will be taken from heavy and light mobile phone users following established methods for cell collection [33]. Reverse transcription-quantitative polymerase chain reaction (RT-qPCR) will be performed on a selection of genes, as defined in in vitro studies with the genomics tool.

**Effects of RF-EMF exposure on human RBCs:** In a first study, blood samples will be taken from healthy volunteers following exposures based on the conditions defined elsewhere in the project (5G carrier frequencies (3G/4G/5G) and modulation envelope). The system will be an evaluation board based on software defined radio (SDR) technology with a maximum frequency of 6 GHz and a minimum quadrature sampling of 8 bits. The output frequency of the multiplier will be emitted in the FR2 band with a modulation scheme as defined in an earlier task. The available output emission will be amplified via a high-frequency amplifier and connected to a directional antenna whose gain will be set according to the required level. A second SDR will be used in reception to ensure the monitoring of the experiment. In a second exploratory study, healthy volunteers (15 males and 15 females aged 18–25 years) will participate in two double-blind sessions, one real and one sham (random) exposure (45 min). In samples taken after exposure, blood count, RBC indices, redox state, and NO level, CO-oximetry, RBC aggregability and morphology will be assessed.

**Biophysical and Biochemical mechanisms of RF-EMF exposure in blood and RBC:** The RF-EMF (4G and 5G) interaction with human RBCs will be studied, in which the possible molecular, sub-cellular/cellular targets of the exposure, and the RF-EMF parameters (repetition frequency, slopes, on-off cycles, etc.) will be identified. A vector signal generator interconnected to an exposure setup equipped with a temperature controller and pumps to introduce flow conditions will be used to expose RBC suspensions. A microfluidic unit will be developed by using a radiofrequency generator and a directional plasmonic phase modulator adapted to 26 GHz. Both systems will allow multiple exposures to RF-EMF signals. A special system will be developed by combining a TEM line for the RF-EMF exposure with a high-performance network analyzer for real-time assessment of dielectric parameters. The responses of cellular hemoglobin (Hb) and membranes will be evaluated using dielectric spectroscopy to monitor changes in protein structure and its surface charge through the changes in free and bound water dynamics. Aggregability, deformability, membrane stability, hydration, and cell morphology will be analyzed using conventional techniques (sedimentation rate, microscopy, the changes in RBC indices) and the methodology available at the partners’ sites (ektacytometry, microfluidic approaches, flow cytometry).

### 3.4. Causal Links between RF-EMF Exposure Level and Duration and Potential Health Effects

The methods for evaluating and integrating the evidence on environment and health topics are transitioning from an “expert-based narrative” to a “systematic” review. SRs of aetiological studies are regarded as the best-practice method for evaluating evidence used in decision-making [34]. Complexity is an inherent feature of environmental health hazard and risk assessments, and methodological advances in all evidence streams relevant to hazard/risk assessments (toxicology, epidemiology, and exposure science) are increasing the challenges for evidence assessment and integration.

**Umbrella review of human cancer hazards from exposure to near-field and far-field sources of RF-EMF:** WHO is undertaking an updated assessment of health hazards from exposure to RF-EMF, including 10 systematic reviews of experimental and observational studies on six major topics (cancer, adverse reproductive outcomes, cognitive impairment, symptoms, oxidative stress, and heat-related effects) [35]. There have already been published detailed systematic review protocols for the effect of RF-EMF on different topics, such as [36,37], which will be considered in NextGEM for setting out research questions and methodology. Cancer is the most relevant potential hazard of RF-EMF at exposure levels below current international guidelines. If RF-EMF exposure increased the risk of cancer, this would have serious public health consequences and require population-level preventive strategies, including a revision of the threshold-based limitation principle currently applied to non-ionizing exposure in the radiofrequency range [38]. Neoplastic diseases are also the most investigated potential adverse effect of prolonged exposure to RF-EMF from mobile communication, accounting for about 56% of all related epidemiological studies (n = 327) up to 2021, as indexed in the specialized literature database EMF-Portal (https://www.EMF-portal.org/en (accessed on 1 March 2023)). NextGEM’s consortium includes cutting-edge methodological and subject-matter expertise in umbrella reviews and meta-analyses required for a critical summary of findings from SRs of epidemiological studies on the potential carcinogenicity of RF-EMF [23], and will focus on this outcome. NextGEM will compile the available evidence on RF-EMF and cancer risk in humans by performing an umbrella review of relevant narrative or quantitative reviews published in the last decade. Umbrella reviews, defined as the systematic collection and evaluation of information taken from multiple systematic reviews and meta-analyses, have the potential to provide the highest quality of evidence, graded according to the criteria described by Papatheodorou [39].

**Development and validation of a Risk Assessment model to assist evidence- informed decision making on RF-EMF exposure:** The basic existing assessments of risks associated with RF-EMF exposure need to be improved, and this is a capital objective of NextGEM. To this aim, relevant data from an exposure assessment, in vitro and in vivo experiments, the level of evidence resulting from the umbrella reviews of human observational studies, the evidence pertaining to experimental studies of carcinogenic effects of RF-EMF in animal and cells models, and the results of the human observational studies conducted within NextGEM will be integrated. Common exposure protocols will be defined at the early stages of this project to ensure the harmonized data collection required for a robust risk assessment.

Based on stakeholder (industry, insurers, regulators, and consumers) input, requirements for output information criteria will be identified. Data underpinning the different stages of risk assessment from different lines of evidence will be obtained from activities and assessed for their appropriateness for hazard identification and characterization, exposure assessment, risk characterization, and uncertainty analysis. The final product is an evidence-based integrative risk assessment. Any lack or shortcoming of data will feed back to activities for completion. The models and output parameters identified in this task will be submitted to a dedicated case study for the first round of sensitivity and performance testing, which, after refinement, will be iterated to involve all case studies.

### 3.5. Development of NextGEM Innovation and Knowledge Hub

**Figure 4:**
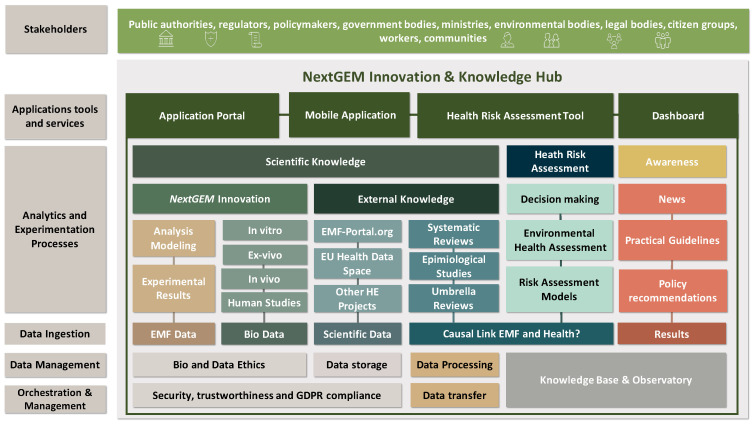
NextGEM innovation and knowledge hub (NIKH).

The implementation of NextGEM Innovation and Knowledge Hub (NIKH) is a practical realization and integration of scientific components. It will monitor, store, share and access EMF exposure and biological data to ensure compliance with safety standards, minimize exposure levels in set environments and contexts, and increase citizens’ awareness on EMF information and research [40]. To enable the validation and exploitation, a benchmarked proof-of-concept reference platform will be developed. The development of NIKH includes four different stages: (i) designing the platform to store the innovations and research outputs produced within NextGEM, (ii) including external scientific knowledge from previous research or through synergies with projects funded under other clusters and pillars of Horizon Europe, or other EU programs, (iii) offering a link to RF-EMF stakeholders, and (iv) enabling security, trustworthiness, and General Data Protection Regulation (GDPR) compliance (Figure 4).

**Platform design for collecting research outputs:** The NIKH will coordinate the processes for translating analytics and experimentation model descriptions to deployed jobs over the end-to-end system from data management and analytics components all the way to end-user applications and services. NIKH aims to offer template services, embodying NextGEM’s logic for diverse research scenarios, managing execution planning (explicit and automatically decided), and handling interactions between NextGEM components while configuring their seamless integration.

**Collection and storing of data within NextGEM and from other projects, activities, and studies**: NextGEM will provide a vast amount of research outputs. The NextGEM workflow includes setting up the RF-EMF data platform, integrating existing data from available public and reviewed sources, and integrating new EMF-relevant data generated within the project. Thus, the stored data will be accessible from the NextGEM dashboard as soon as the primary obligations related to scientific publishing, etc., are fulfilled. NextGEM will furthermore make the data collection accessible and expandable for users after project end, which needs a long-term commitment regarding database management.

**Connection with the RF-EMF Stakeholders:** In the multimodal environment, NIKH will provide a fully configurable NextGEM dashboard for public authorities, the scientific community, and various citizen and societal groups. A graphical web-based front-end dashboard will be developed to facilitate the visualization of data from RF-EMF measurements, analytical models, bio-experiment results, and risk assessment together with practical guidelines, tools, and applications, thus supporting public authorities and regulators with scientific evidence to implement exposure directives and improve their RA, management and communication.

**Security, trustworthiness and GDPR compliance:** To assure NextGEM operation from a cybersecurity perspective, as well as to provide evidence for its GDPR compliance, the SANL Security Assurance Platform (SAP) will be employed [41]. Following a model-driven approach, the SAP will be tailored to the NIKH. The real-time operational compliance of the platform to security, privacy, and GDPR requirements will be provided in an evidence-based manner through the integration of the SAP, which will enable hybrid assessments based on vulnerability analysis, penetration testing, and continuous monitoring of the NextGEM assets. This establishment and operation of continuous security and privacy assurance checks for the NIKH and its security and privacy control mechanisms will ensure the security and privacy posture of it.

## 4. Case Studies

NextGEM results will be applied and validated through three case studies, covering different groups of geographical and socio-economic conditions, as well as vulnerable people exposed to different signals (i.e., multiple RF-EMF exposure, including 4G and 5G signals, focused on FR1 or FR2): (1) potential effects of indoor levels of RF-EMF exposure of vulnerable people on reproduction and development, (2) optimised outdoor urban planning and 5G design architecture and investigations for public awareness of cancer-related health hazards, and (3) bealth effects of exposure to RF-EMF in indoor and outdoor environments.

### 4.1. Case Study 1—Potential Effects of Indoor Levels of RF-EMF Exposure of Vulnerable People on Reproduction and Development

**Figure 5:**
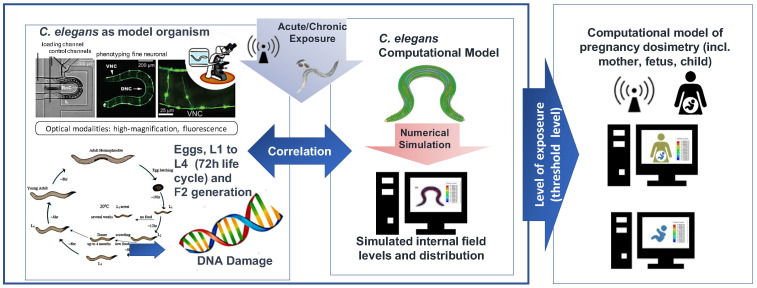
Case Study 1—Potential effects of indoor levels of RF-EMF exposure of vulnerable people on reproduction and development.

The COVID-19 pandemic has increased the general public’s indoor exposure to RF-EMF radiated by multiple sources, such as wireless personal communication devices (Wi-Fi, Bluetooth) or other applications (security scanners, smart meters, and medical equipment) in conjunction with ambient environmental exposure. The effect of RF-EMF exposure on the health of vulnerable people, such as pregnant women or children, has not been studied sufficiently. In addition, the potential exposure to RF-EMF and other physical or chemical agents has increased concerns regarding fertility and children’s development. The main scope of NextGEM in Case Study 1 (Figure 5) is to investigate potential RF-EMF effects on vulnerable people by analyzing the existing literature and developing an exposure modeling approach to establish possible thresholds for safe/unsafe situations of single and multiple exposures and the potential risk on fertility and children’s development.

To this aim, the review of scientific evidence on RF-EMF and adverse reproductive outcomes will be undertaken. Appraisal of the evidence regarding potential hazards of RF-EMF exposure for pregnant women and children (health agencies, risk assessors) is an important and often overlooked issue for the community. Therefore, the field distribution will be simulated numerically inside the body of fetuses, pregnant women, and children (ERMES software).

In addition, *C. elegans* will be evaluated as a simple in vivo organism fulfilling 3R requirements. The collection of electromagnetic property values of *C. elegans* tissues and their growth medium will be obtained from own measurements and the literature. The exposure of *C. elegans* to different RF-EMF frequencies and modulation patterns, along with simultaneous numerical simulations, will be carried out to obtain the field distribution in critical parts of the body (eggs, reproductive system). The potential cross-generational health effects on reproduction and development will be investigated experimentally during the life cycle of *C. elegans* after RF-EMF exposure. The results of both numerical simulations and *C. elegans* experiments performed will be evaluated separately and fed into the models.

### 4.2. Case Study 2—Optimised Outdoor Urban Planning and 5G Design Architecture and Investigations for Public Awareness on Cancer-Related Health Hazards

**Figure 6:**
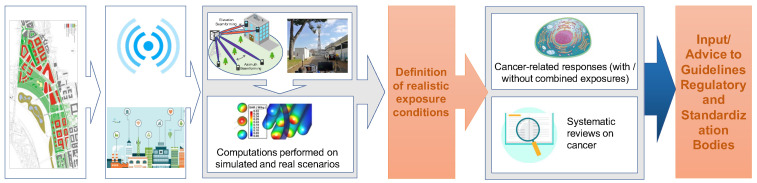
Case Study 2—Optimized outdoor urban planning and 5G design architecture and investigations for public awareness on cancer-related health hazards.

5G is considered the key technology for enabling a wide range of application scenarios and the effective spreading of the smart city concept. In total, 50 billion devices are foreseen in such a scenario by 2023, with a connection density of 1 million/km${}^{2}$ (https://www.accenture.com/_acnmedia/PDF-146/Accenture-5G-WP-US.pdf (accessed on 1 March 2023) spread over different outdoor and indoor locations, from underground to sky coverage for unmanned aerial vehicle (UAV) applications. Moreover, the use of Ma-MIMO antennas and higher frequency bands has completely changed the network’s coverage and management requirements. RF-EMF will only be present when and where it is needed. In parallel to measurements, computations will be performed on simulated and real scenarios to analyze the effect of Ma-MIMO antennas and transmitter positions on field distribution. However, there are concerns that this technology increases the risks of carcinogenesis. The scope of this case study (Figure 6) is to assess the urban planning and exposure management for 5G NR location design architecture and to examine the possibility of cell site distribution in an urban environment to optimize the exposure and to analyze field distribution over the territory due to Ma-MIMO antennas.

Real standard measurement periods, in compliance with ICNIRP guidelines, and long-term (hours to days) acquisitions will be carried out to evaluate 5G RF-EMF exposure by resorting to maximum power extrapolation (MPE) techniques applied to the field level measured in real traffic and environmental conditions, both in line-of-sight and non-line-of-sight conditions. Statistical models will be developed and validated in real scenarios to consider such variability in time and space of the distribution, coverage, and design antenna mapping. In this respect, the measurement procedures for coverage quality evaluation and exposure assessment need to be rethought and reviewed to implement effective and efficient measurement methodologies for validating developed models in optimal planning. Urban planning, based on ray-tracing methodologies, will be performed on simulated and real scenarios to analyze transmitter positioning on field distribution and levels. The RF-EMF field distribution over scenarios due to the new technologies (e.g., 5G and Ma-MIMO) and such statistical models will be evaluated in terms of the exposure characterization for different purposes, such as new methodologies for exposure assessment.

In addition, biological investigations will be carried out in vitro (neuroblastoma cells and human keratinocytes) and ex vivo (human lymphocytes) to gain information on potential health risks under realistic exposure conditions in 5G frequencies as used in existing 5G design architectures. Selected realistic conditions will be tested to investigate biological responses in the presence and absence of other agents (combined exposures). An umbrella review of epidemiological studies of cancer risks in relation to near-field and far-field RF-EMF exposure and risk assessment approaches on cancer will be performed.

### 4.3. Case Study 3—Health Effects of Exposure to RF-EMF in Indoor and Outdoor Environments

**Figure 7:**
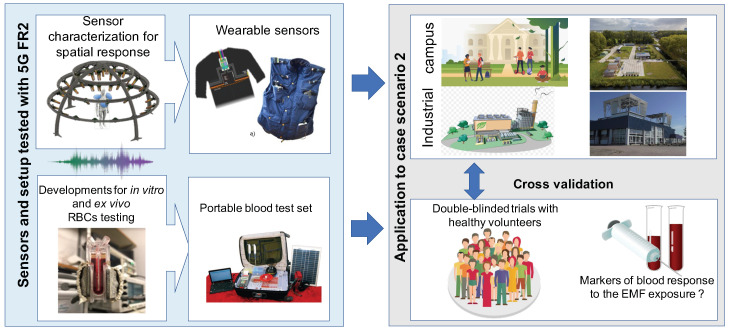
Case Study 3—Health effects of exposure to RF-EMF in indoor and outdoor environments.

The 5G system is expected to enable smart industries by providing massive and reliable high-bandwidth communication links and creating high-bandwidth campus environments supporting high data rate connectivity. 5G wireless networks will employ higher frequency bands to support such connectivity demands. In such scenarios, the RF-EMF exposure in specific locations will be highly dynamic in time and space, based on the user’s data request and spatial movement in a given area. The passive RF-EMF exposure of individuals will then vary considerably compared to 4G/LTE cases. Users with different demands in the same workplace are exposed to various sources of RF-EMF. However, the adoption of the high-frequency band, and usage of beam steering devices, in both indoor and outdoor locations (e.g., campus, industrial) requires the development of novel measurement devices and deeper analysis to evaluate the potential health risks or symptoms due to the directional RF-EMF exposure in high frequencies (FR2, above 26 GHz). The main scope of this case study (Figure 7) is to assess the exposure of 5G signals in the FR2 mmWave bands according to personalized demands in indoor and outdoor environments and studies of possible biological effects and their mechanisms on RBC.

To achieve this goal, NextGEM will conduct an extensive literature review on the effects of RF-EMF biophysical mechanisms and on RF-EMF assessment sensing technology. The indoor and outdoor exposures will be modeled using the ERMES ray-tracing tools, including the provision of a first-order model including scattering from the environment to computed received power. This case study will define the indoor (i.e., industrial environment) and outdoor university campus environment with multiple FR2 antenna modules with steering capabilities covering the defined area to provide a higher data rate. This includes identifying signal test cases/scenarios to identify low-, medium-, and high-body RF-EMF exposure.

The assessment of real-life exposure will be measured with wearable vest sensors. The sensing element will provide the required sensitivity to accurately sample the average energy received in the selected case study, operating in the FR2 mm wave bands and capable of covering the entire-body RF-EMF exposure (i.e., distributed sensors). The number and the location of the developed sensors on the vest will provide entire-body RF-EMF exposure. The testing of measurement systems will be carried out in the TU Delft lab and the outdoor measurement in the IoT Green Village 5G in the Delft Campus with the participation of volunteer students. In addition, the possible biophysical and biochemical mechanisms of mmWave exposure (FR2) will be investigated on RBC to identify the fundamental mode of action of acute (minutes to hours) exposure and to identify the signal features (pattern, intensity, duration) of importance. If the in vitro experiments show effects of the exposure found in the case study, a double-blind experiment on volunteers will be performed to investigate the possibility of blood effects.

### 4.4. Expected Outcomes from Case Studies

It is expected that the case studies will provide different outcomes to the scientific society, citizens, public authorities and the regulator. More specifically, Case Study 1 can upgrade simulation tools and models (useful for exposure assessors) which the scientific community can apply in further research on RF-EMF exposure. In addition, health standards will be updated regarding the potential effects of different frequencies (health agencies, risk assessors) and, finally, the outcome of the research will generate the basis for practical guidelines. The expected outcome of Case Study 2 will include the antenna optimization mapping criteria (telecom operators) and updating or verifying RF-EMF limits (regulators). It will also increase knowledge and public awareness regarding the biological responses and 5G frequency exposures. The expected outcome of Case Study 3 will include the assessment of actual exposure to 5G frequency bands in everyday activities on the campus. Finally, the identification of a potential correlation between 5G RF-EMF exposure parameters and RBC effects (health agencies, risk assessors) will be useful to increase the knowledge and awareness of citizens regarding potential health issues and 5G.

## 5. Conclusions

The ambitious vision of NextGEM was presented in this work through the description of the specific activities and related methodologies. The multidisciplinary expertise of the NextGEM partnership spanning over telecommunication engineering, cell biology, human studies and epidemiology, will be exploited during the four-year project to guarantee the fulfillment of its main objectives. Relevant scientific knowledge and data on new scenarios of the next generation of RF-EMF, on the effects and interaction with biological materials relevant to human health, will be generated in the project. Together with the current epidemiological knowledge on cancer and reproductive outcomes, this knowledge will be integrated into an evidence-based risk assessment for use by different stakeholders. The development of the NextGEM Innovation and Knowledge Hub for EMF and Health will ensure the accessibility of the project results to the scientific community, the public, and the European regulators and improve awareness. Finally, NextGEM results will be validated in three case studies to ensure the secure and safe living of vulnerable citizens and workers.
